# Integrating DNA/RNA microbe detection and host response for accurate diagnosis, treatment and prognosis of childhood infectious meningitis and encephalitis

**DOI:** 10.1186/s12967-024-05370-w

**Published:** 2024-06-20

**Authors:** Zhihao Xing, Hanfang Jiang, Xiaorong Liu, Qiang Chai, Zefeng Xin, Chunqing Zhu, Yanmin Bao, Hongyu Chen, Hongdan Gao, Dongli Ma

**Affiliations:** 1 Biobank & Clinical laboratory & Department of Respiratory Medicine, Shenzhen Children’s Hospital of Shantou University Medical College, Shenzhen, Guangdong China; 2 Institute of Pediatrics, Shenzhen Children’s Hospital, Shenzhen, Guangdong China; 3 Clinical laboratory, Shenzhen Children’s Hospital, Shenzhen, Guangdong China; 4 Department of Respiratory Medicine, Shenzhen Children’s Hospital, Shenzhen, Guangdong China; 5 Medical Testing, Bengbu Medical College, Bengbu, Anhui China

**Keywords:** Infectious meningitis and encephalitis, mNGS, Pathogen, Host response, Antibiotic resistance, Prognosis

## Abstract

**Background:**

Infectious meningitis/encephalitis (IM) is a severe neurological disease that can be caused by bacterial, viral, and fungal pathogens. IM suffers high morbidity, mortality, and sequelae in childhood. Metagenomic next-generation sequencing (mNGS) can potentially improve IM outcomes by sequencing both pathogen and host responses and increasing the diagnosis accuracy.

**Methods:**

Here we developed an optimized mNGS pipeline named comprehensive mNGS (c-mNGS) to monitor DNA/RNA pathogens and host responses simultaneously and applied it to 142 cerebrospinal fluid samples. According to retrospective diagnosis, these samples were classified into three categories: confirmed infectious meningitis/encephalitis (CIM), suspected infectious meningitis/encephalitis (SIM), and noninfectious controls (CTRL).

**Results:**

Our pipeline outperformed conventional methods and identified RNA viruses such as Echovirus E30 and etiologic pathogens such as HHV-7, which would not be clinically identified via conventional methods. Based on the results of the c-mNGS pipeline, we successfully detected antibiotic resistance genes related to common antibiotics for treating Escherichia coli, Acinetobacter baumannii, and Group B Streptococcus. Further, we identified differentially expressed genes in hosts of bacterial meningitis (BM) and viral meningitis/encephalitis (VM). We used these genes to build a machine-learning model to pinpoint sample contaminations. Similarly, we also built a model to predict poor prognosis in BM.

**Conclusions:**

This study developed an mNGS-based pipeline for IM which measures both DNA/RNA pathogens and host gene expression in a single assay. The pipeline allows detecting more viruses, predicting antibiotic resistance, pinpointing contaminations, and evaluating prognosis. Given the comparable cost to conventional mNGS, our pipeline can become a routine test for IM.

**Supplementary Information:**

The online version contains supplementary material available at 10.1186/s12967-024-05370-w.

## Introduction

Infectious meningitis/encephalitis syndromes (IM) are severe neurological infectious diseases caused by bacterial, viral, and fungal pathogens, with higher diagnostic error and high morbidity, mortality, and sequelae in childhood. IM mainly included bacterial meningitis (BM) and viral meningitis/encephalitis (VM) [1]. BM is one of the common infectious diseases in children, especially newborns, and 20–50% of newborn survivors may have sequelae [2]. Most clinical symptoms of IM are not specific. In the absence of etiological diagnosis, noninfectious syndromes that resemble IM further complicate diagnosis and confound targeted treatment. Accurate detection of pathogens in IM patients and identifying patients with poor prognoses is crucial for prompt and adequate targeted treatment [3].

However, laboratory microbiologic detections are limited by available microbiologic tests because cerebrospinal fluid (CSF) culture is less sensitive and time-consuming, and CSF PCR can only target several pre-defined microbes, which will lead to the empirical use of broad-spectrum antibiotics, which highlights the need for less restricted methods, such as metagenomic sequencing (mNGS). The mNGS can enable unbiased detections of all potential pathogens and especially performs well in identifying difficult-to-culture, rare and novel pathogens [4]. Some studies have used mNGS to diagnose infectious central nervous system (CNS) diseases [5, 6]. However, routine mNGS pipelines are mainly developed for DNA microbes, and another RNA library construction is necessary to sequence RNA viruses, which will need extra time and cost [7, 8]. Thus, simultaneous detections of RNA and DNA microbes in one mNGS workflow is an essential requirement for routine clinical pathogen detection for infectious encephalitis/meningitis (IM). Sander et al. and Bal et al. developed an mNGS protocol for routine DNA and RNA viral respiratory infection diagnostics with sensitivity comparable to PCR [9, 10].

We have developed an mNGS protocol that could enable the detection of DNA and RNA pathogens in the Cerebrospinal fluid (CSF) samples [11], and the performance in the diagnosis of IM should be studied using clinical samples. Besides, our mNGS protocol further provided one additional potential in evaluating the host transcriptional profiling by mNGS. Host transcriptional profiling has emerged as a promising alternative to pathogen-based diagnostics that can identify respiratory infections from those with noninfectious illnesses [12–14]. It also performed well in evaluating the prognosis of respiratory infections and sepsis [15, 16]. Furthermore, host transcriptional profiling has been coupled with the simultaneous detection of pathogens to improve the diagnosis of tuberculosis meningitis and acute respiratory infections [17, 18].

However, while highly promising, this approach has not been well studied in IM. This study may extend current etiological diagnostics and treatments by detecting pathogens and host transcriptional profiling simultaneously. Firstly, we evaluate the performance of this approach in a large retrospective cohort of IM patients in Shenzhen Children’s Hospital, the only sentinel pediatric hospital in Shenzhen, covering more than 250 IM patients annually. Moreover, we address the need for better diagnostics, antibiotic resistance prediction, contamination discrimination, and prognosis of IM by integrating host response and DNA/RNA microbe detection. These results may provide an important basis for diagnosing and treating IM.

## Method

### Samples collection and analysis

We retrospectively reviewed the CSF samples at Shenzhen Children’s Hospital to identify infectious meningitis/encephalitis (IM) samples. Uninfected samples of leukemia and other diseases were used as controls. All patients underwent standard microbiologic diagnostics testing, and the retention times of CSF samples are the same as corresponding tests. Physicians identified subjects with CSF without knowing the mNGS results. Patient characteristics (age and gender), clinical treatments, clinical laboratory indicators, and prognosis were extracted from the hospital database. All clinical and laboratory features were obtained the same day the mNGS test occurred. 142 samples were finally included and grouped into three categories according to clinical and microbiologic observations:

#### Confirmed infectious meningitis/encephalitis (CIM)

The clinical diagnosis was BM or VM, and CSF PCR or culture test was positive for the bacterial or viral pathogen.

#### Suspected infectious meningitis/encephalitis (SIM)

1. Clinical diagnosis supported BM or VM, but CSF culture and PCR test were negative.
2. Or clinical diagnosis was uncertain but CSF culture or PCR test was positive.

#### Noninfectious samples (CTRL)

Clinical diagnosis was leukemia and other diseases without infectious symptoms, and CSF culture and PCR test were negative.

Microbes identified by clinician-ordered diagnostics in the 36 CIM samples positive for PCR target viruses and culture-dependent bacteria were categorized as pathogens (*n* = 28 in the training cohort and *n* = 8 in the validation cohort). We accepted that this practical gold standard would provide an attenuated estimate of performance due to the sensitivity limitations of microbial culture in the setting of antibiotic pre-administration [17].

### Total nucleic acid extraction

Following our previous method [11], total nucleic acids (RNA and DNA) were extracted with the EasyPure RNA Kit (TransGen, China), and RNA was reverse-transcribed using the Transcriptor first-strand cDNA synthesis kit (Roche, Switzerland). The second strand cDNA synthesis was achieved using NEBnext mRNA Second Strand Synthesis Module (NEB, USA). The average DNA yield is 0.9 µg, as quantified using the qubit and qPCR.

### Next-generation sequencing

Finally, both cDNA and gDNA (genomic DNA) were used for library generation and sequencing. This method consisted of a single nucleic acid extraction step and a single library generation and combined and integrated the advantages of both RNA-seq and WGS, which can measure both DNA/RNA pathogens and host gene expression in a single assay. Sequencing was performed by Novogene Inc (Tianjin, China) on the Hi-seq 2000 sequencing system (Illumina), generating 2 × 150 paired-end reads.

### Taxonomic classification

Kraken2 pipeline was used for taxonomic classification and abundance quantification at the species level as in our previous study [11, 19]. First, low-quality bases (q ≤ 30) and adapter sequences were trimmed using Trimmomatic v0.36 with default parameters [20]. The reads with fewer than 36 (samples with bacteria) or 140 (samples with viruses) bases were filtered out. Accordingly, 1.32 billion clean paired-end reads were obtained across the 142 samples, with an average of 9.3 million reads per sample (Supplementary Table 1). Second, Kraken2 was used to taxonomically classify the clean reads with default parameters [19]. Kraken2 had similar, and often superior, per-sequence accuracy to other classifiers with high processing speed and fewer memory requirements. Kraken2 maps and classifies overlapping 31-kmer bp sequences to the most recent common ancestor to provide the most accurate taxonomic classification, such as species and genus. The default reference databases for Kraken2 were built from RefSeq bacteria, archaea, viral libraries, and the GRCh38 human genome. By including the GRCh38 human genome in the reference database, Kraken2 allows for easy and accurate classification and removal of human reads [19]. The outputs of Kraken2 were visualized by Pavian v1.0 [21].

### Definitions and calculation formula

The positive cutoff of non-viral pathogens referred to the previous reports [11] and 10 samples from the CTRL group were used as negative CSF samples (NCSF). If microorganisms were not detected in the negative cerebrospinal fluid (NCSF), the RPKM in NCSF was set to 1, and the RPKM ratio (RPKMratio) was calculated. The positive viral cutoff was three noncontiguous or non-overlapping fragments of more than 140 bp on the genome covered [11], and the viral species did not exist in the NCSF. The coverage was displayed by Integrative Genomics Viewer (IGV 2.8.10).

Raw reads (RR): Refers to the number of reads classified to a specific species.
Genome size (GS): Refers to the genome size of the microorganism’s genome (Mb).
Total reads (TR): Refers to the reads classified as microorganisms.
RPKM: The value of RR/(GS*TR).
RPKM_SAMPLE_: The RPKM of a certain microorganism in the CSF samples.
RPKM_NCSF_: The RPKM of a certain microorganism in NCSF.
RPKMratio: The ratio of RPKM_SAMPLE_ to RPKM_NCSF_.

### Analysis of antibiotic resistance genes by mNGS

All mNGS reads were searched for antibiotic resistance genes (ARGs) using UBLAST (with E-value ≤ 10 − 7) against the curated structured ARG database SARG, which integrates ARDB, CARD, and the latest NCBI-NR databases [22]. When an alignment with length ≥ 75 nucleotides and identity ≥ 80% was found [22, 23], the hit ARG was deemed found in a sample. The abundances of ARGs were normalized using UBLAST with default option [22, 23]. The heatmap plot for ARGs was conducted using the R pheatmap package.

### Transcriptional analysis of host genes

RNA reads were aligned to the UCSC human hg19 reference genome using STAR [24]. The read counts for each gene were summarized using the program featureCounts, requiring counted reads uniquely aligned and ≥ 90% matched to gene [25]. The read count matrix from featureCounts was inputted into the Bioconductor package DESeq2 to identify differentially expressed genes [26] with the cutoffs FDR < 0.05 and |Log_2_FC| >0.5. The normalized counts generated by DESeq2 were used in the following analysis.

### KEGG pathway GSEA enrichment analysis

Functional enrichment analyses of BM and VM-related genes were conducted using the Kyoto Encyclopedia of Genes and Genomes (KEGG) via R package clusterProfiler [27]. FDR < 0.05 was the cutoff criteria to identify the enriched KEGG pathways.

### Construction of BM classification model

#### Gene feature selection

Using the matrix of read counts per gene as input, we selected differentially expressed gene features between BM and CTRL samples via the DaMiRseq package [28]. Covariates such as batches, age, and gender were included in the model to reduce the effect of irrelevant sources. Differentially expressed features were used as predictors for the below classification model.

#### Construction of the classification model

We used Lasso logistic linear regression (cv.glmnet function in R glmnet package) to reduce complexity and overfitting and build the classification model [29]. The formula is as follows:

$$\omega ={\text{a}\text{r}\text{g}\text{m}\text{i}\text{n}}_{\omega }\left(\sum {(Y-{\omega }^{T}X)}^{2}+\lambda \left|\right|\omega \left|\right|\right)$$

X refers to the expressions of differentially expressed genes selected via DaMiRseq. Y refers to the classification of samples, including BM and CTRL samples. The parameter λ controls the overall strength of the penalty. λ is estimated by cross-validation and the recommended lambda.1se (largest value of lambda such that error is within one standard error of the minimum lambda) is used to choose the simplest model whose accuracy is comparable with the best model [29].

### Construction of the BM prognosis model

The BM samples with prognosis information were randomly divided into training (*n* = 33) and test datasets (*n* = 18). Training datasets are used to build the model, and test datasets are used to assess the possible future performance of the BM prognosis model. Similar to the above classification model, Lasso logistic linear regression model (cv.glmnet function in R glmnet package) was used to train the BM prognosis model:

$$\omega ={\text{a}\text{r}\text{g}\text{m}\text{i}\text{n}}_{\omega }\left(\sum {(Y-{\omega }^{T}X)}^{2}+\lambda \left|\right|\omega \left|\right|\right)$$

X refers to the expressions of poor prognosis-related genes obtained from DaMiRseq. Y refers to the outcome of the samples and includes two groups (good and poor prognosis). Cross-validation is used to estimate the parameter λ and the recommended lambda.1se value is chosen in the final tuned model.

### ROC curve

ROC curves were generated to evaluate the models’ performance using the R packages ggplot2 and pROC [30]. Sensitivity and specificity were calculated using the R ROCR package [31].

## Results

### The performance of the comprehensive mNGS protocol

**Fig. 1:**
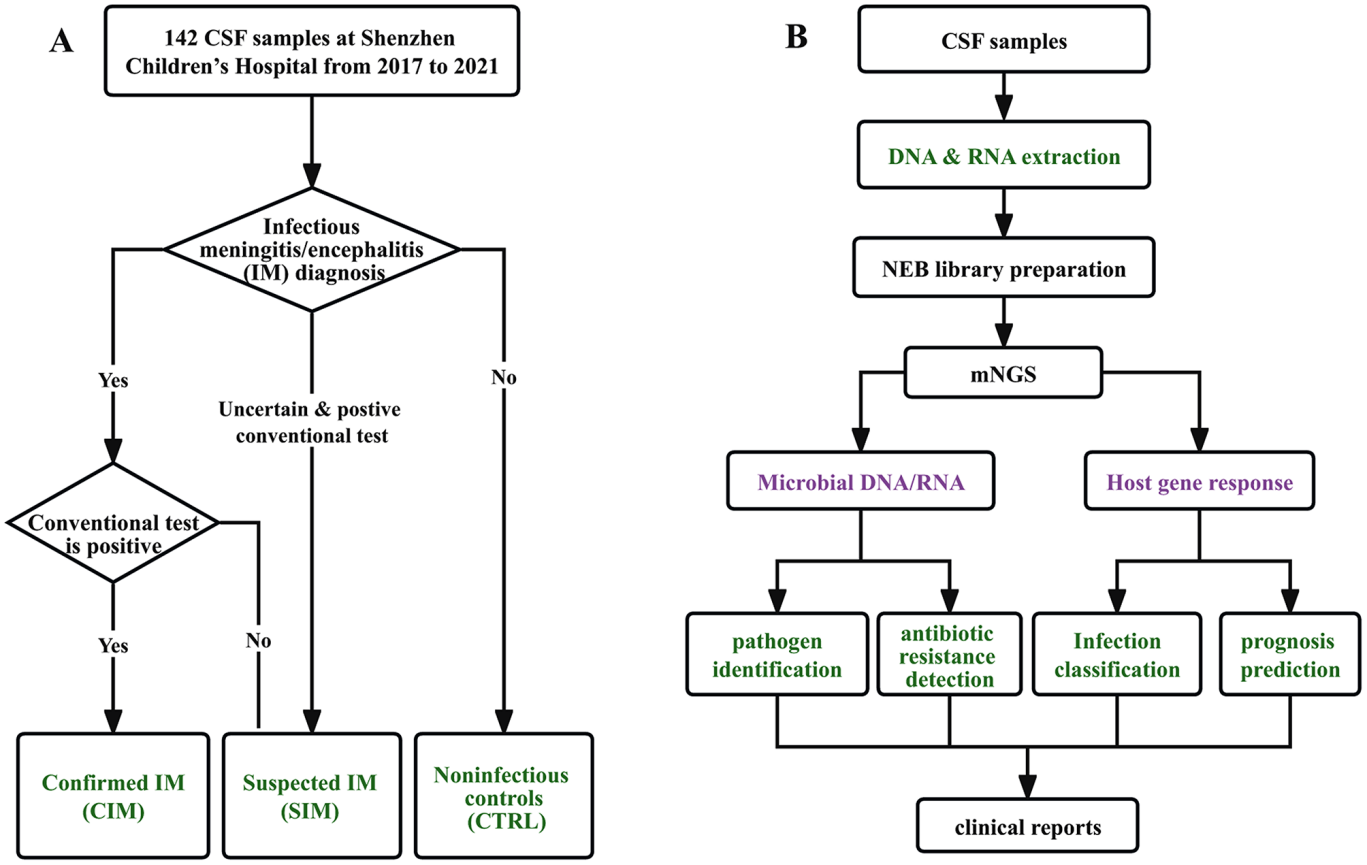
The workflow of this study. (**A**) The groups of samples involved in this study. (**B**) The workflow of the study

In our previous study, we developed an mNGS protocol (comprehensive mNGS, c-mNGS), allowing the detection of both DNA and RNA pathogens (including DNA viruses, RNA viruses, G + bacteria, and G- bacteria) in the samples of infectious meningitis/encephalitis (IM) in a single assay, which reduces the cost and turnaround time compared to the conventional mNGS protocols that target DNA and RNA separately [11]. In this study, we tested the performance of this protocol using a large number of CSF samples from IM patients (Fig. 1A). Briefly, 142 samples were included in this study, which were divided into three groups: CIM (*n* = 36), SIM (*n* = 43), or CTRL (*n* = 63) (Fig. 1A) based on traditional microbiological tests.

**Fig. 2:**
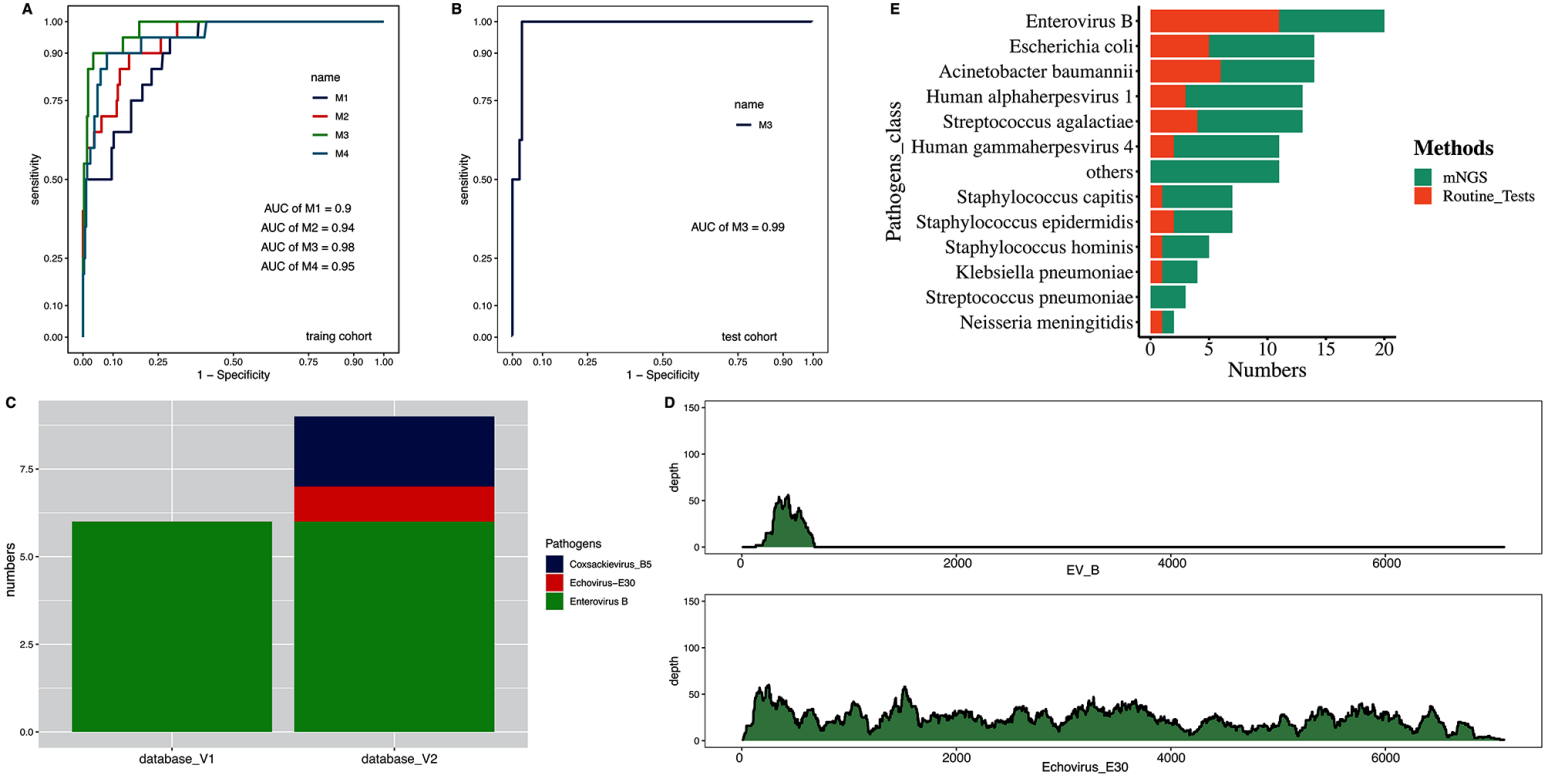
Establishment of mNGS pipeline for pathogen detections. (**A**) The comparisons of several different normalization methods for pathogen detections in training cohorts. M1: total reads are used as normalization, M2: Total micro reads are used as normalization, M3 (RPKMratio): total micro reads and genome size are used as normalization, M4: RPKMratio: total micro reads and genome size are used as normalization without PCR duplicate removal. (**B**) The preformation of RPKMratio-based normalization in the validation cohort. (**C**) Enterovirus detected by raw database and updated database. (**D**) The genome coverage of Echovirus E30, which is detected by updated database only. (**E**) The pathogens detected by routine tests and mNGS in the same infectious meningitis and encephalitis cohorts, respectively

For these samples, we generated an average of 9.30 million paired-end reads per sample (Supplementary Table 1). The kraken2 pipeline was used to align reads and identify microbial taxa as in our previous study [11, 19]. The average microbial reads from each sample was 69,082 (Supplementary Table 1). The abundance of each microbe in a sample was estimated using RPKM_ratio_ values (See methods). We optimized both methodology and pathogen reference databases to improve the accuracy of taxa identification. Using the RPKM_ratio_ as the abundance estimate and the updated reference database, our protocol can achieve AUC = 0.98 and 0.99 in the training and testing data, respectively (Fig. 2A and B). Our protocol also detected 50% more enteroviruses than the conventional protocols (Fig. 2C and D). Using RPKM_ratio_>9.134 as the cutoff for positive prediction, our protocol can reach sensitivity and specificity of 90% and 96.6% in the training samples and 100% and 92.9% in the testing samples.

Additionally, our protocol can detect infections in the SIM samples where pathogens were not detected with culture or PCR in routine diagnostics (Fig. 2E). For example, HHV-7 virus was detected with 42 reads in one sample. Further, several bacterial pathogens were detected in some SIM samples with negative culture results, mainly Streptococcus pneumoniae and Ureaplasma parvum. All the newly detected pathogens using our protocol are listed in Supplementary Table 2.

### Antimicrobial resistance genes of bacterial pathogens detected by mNGS

The mNGS also provides a portfolio of potential antibiotic resistance genes (ARGs) for bacterial pathogens, enhancing bacterial diagnostics and guiding treatments, and improving antibiotic stewardship [32–35].

To evaluate ARG prediction by our mNGS protocol, we started with 24 IM-positive CSF samples with > 1000 reads for bacterial pathogens. Overall, the identified ARGs are mainly associated with beta-lactam, aminoglycoside, multidrug, tetracycline, and polymyxin (Supplementary Fig. 1) and are highly heterogeneous over samples. For example, multidrug-resistant genes account for most reads in most samples. In contrast, aminoglycoside-resistant genes and beta-lactam-resistant genes accounted for more than 50% of reads in some other samples (Supplementary Fig. 1).

To check whether the identified ARGs in a sample can predict the bacteria’s antibiotic resistance, we used antimicrobial susceptibility testing as the gold standard and considered the samples with detection of Acinetobacter baumannii (AB, 5 samples), Escherichia coli (E. coli, 8 samples), and Streptococcus agalactiae (GBS, 5 samples) as these pathogens are most frequent.

**Fig. 3:**
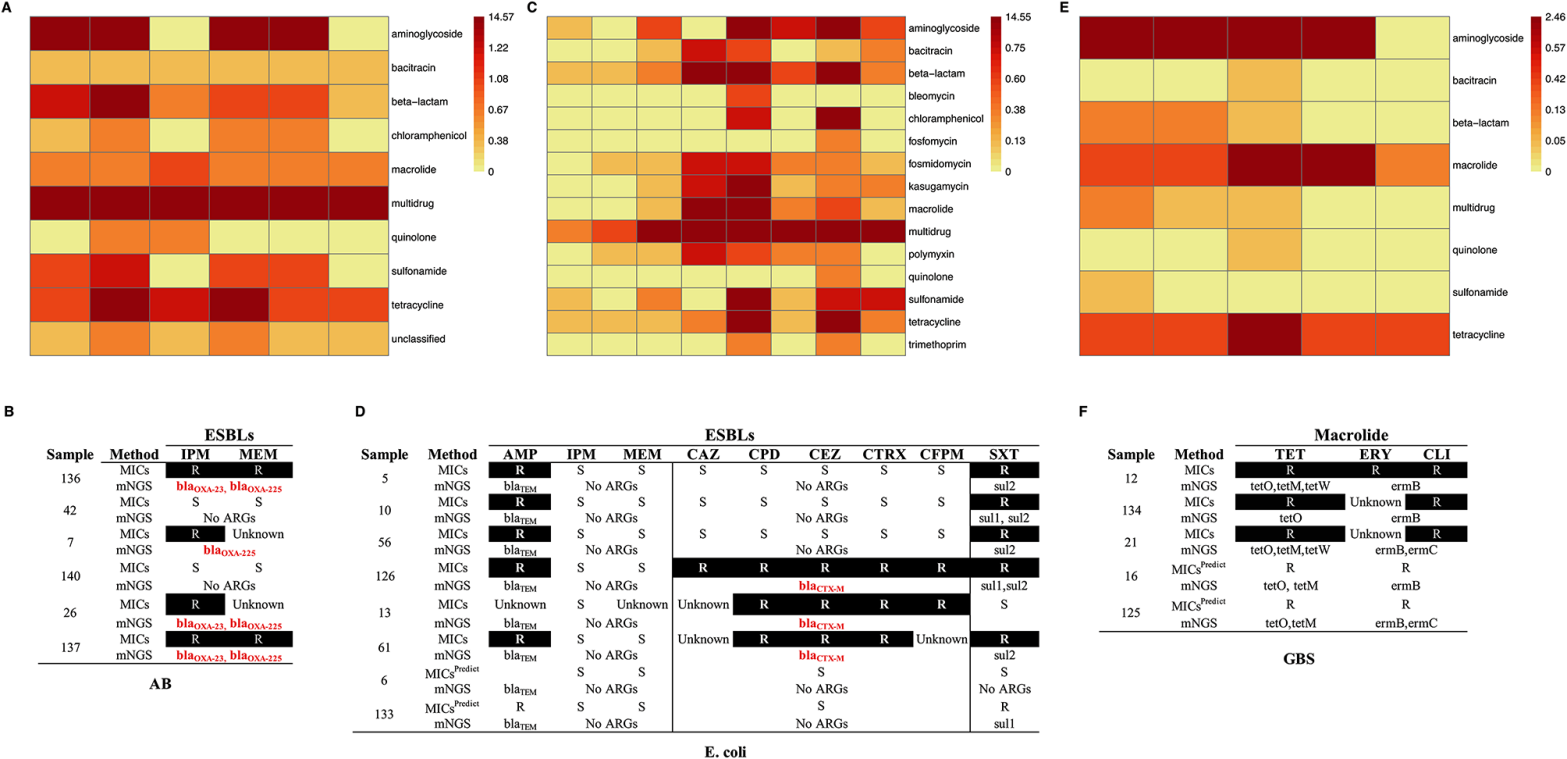
Detections of ARGs in AB, E. coli, and GBS. (**A**) The ARG types and consistency of ARGs with antimicrobial susceptibility testing for AB (**A**-**B**), E. coli (**C**-**D**), and GBS (**E**-**F**), respectively. The full names of the antibiotics abbreviated in the figure are as follows: IPM (Imipenem), MEM(Meropenem), AMP (Ampicillin), CAZ(Ceftazidime), CPD (Cefpodoxime), CEZ (Ceftizoxime), CTRX (Ceftriaxone), CFPM (Cefepime), SXT (Sulfamethoxazole), TET (Tetracycline), ERY (Erythromycin) and CLI (Clindamycin)

In the AB samples, most ARGs are associated with antibiotics like extended-spectrum β-lactamase (ESBLs), aminoglycoside, and multidrug antibiotics (Fig. 3A). For example, bla_OXA−23_ and bla_OXA−225_ genes are found in most samples, and their presences predict resistance to commonly used ESBLs (such as IPM and MEM) (Fig. 3B). Similarly, in the E.coli samples, the presence of the ARG CTX-M co-occurs with the resistance to cephalosporin antibiotics (Fig. 3C-D). In all 3 GBS samples (12, 21, and 134) with susceptibility tests, the presence of ARGs ermB and ermC predicts resistance to Macrolide, and the presence of tetO, tetM, and tetW predicts resistance to tetracycline (Fig. 3E–F).

### Host response genes in infectious meningitis/encephalitis

Exploring the host responses can provide insights into both the diagnosis and prognosis of IM. Our c-mNGS protocol measures both DNA and RNA at the same time and thus provides the ability to profile host gene expressions (Fig. 1B).

**Fig. 4:**
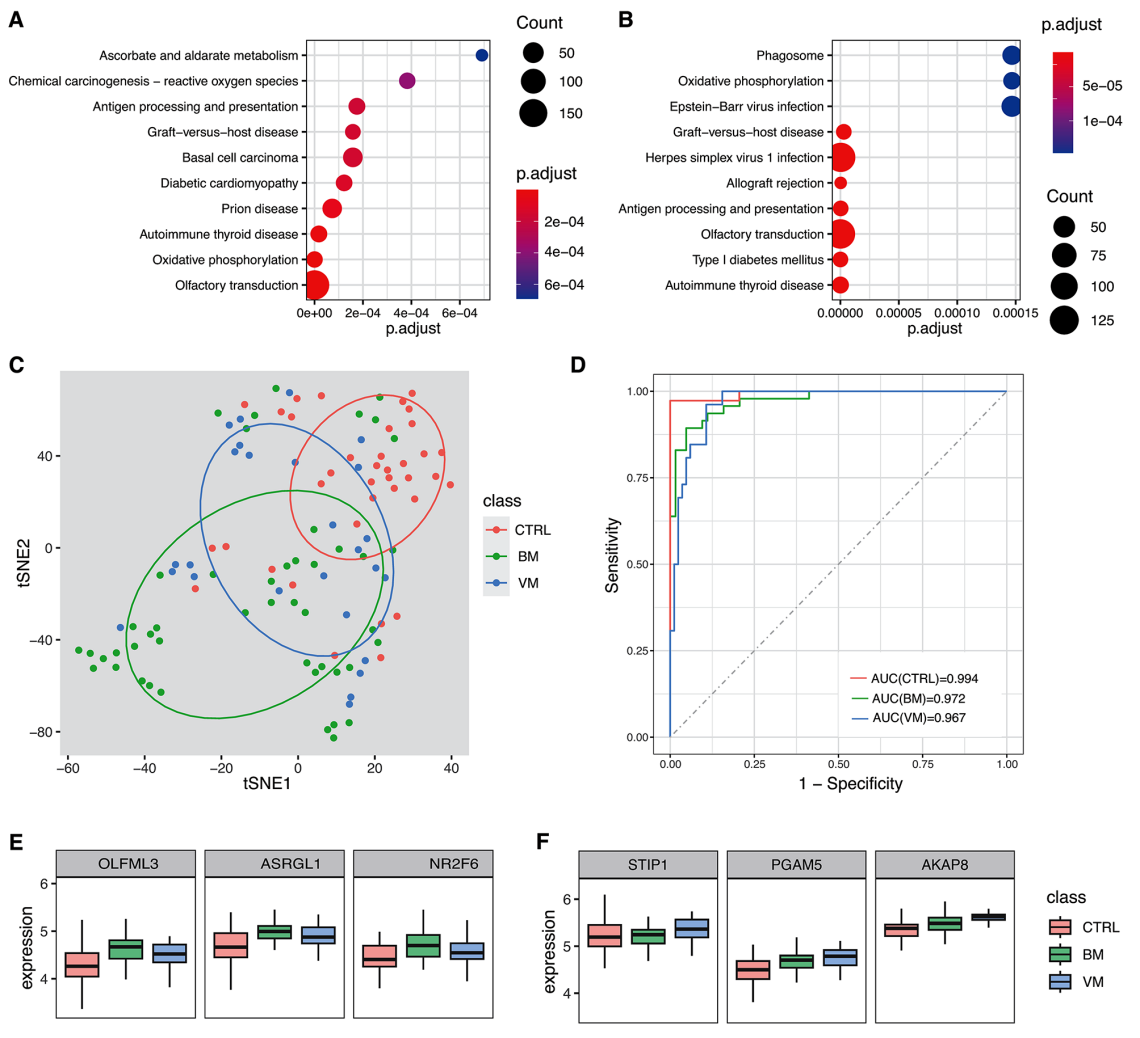
Functional enrichment analysis, classification model, and biomarkers for IM. (**A**) The enriched KEGG pathways between BM and CTRL samples. (**B**) The enriched KEGG pathways between VM and CTRL samples. (**C**) The t-SNE visualization results for BM, VM, and CTRL. (**D**-**E**) The expressions of BM-associated genes (**D**) and VM-associated genes (**E**)

To identify differentially expressed genes, we compared the samples of bacterial meningitis (BM; 47 samples) and the control (CTRL; 37 samples). We identified 1036 DEGs (Supplementary Table 3) and found 48 enriched KEGG pathways by the GSEA method. The top terms include oxidative stress (hsa00190: Oxidative phosphorylation and hsa05208: Chemical carcinogenesis-reactive oxygen species) and antigen processing (hsa04612: Antigen processing and presentation) and immune responses (hsa05332: Graft-versus-host disease and hsa05320:Autoimmune thyroid disease) (Fig. 4A).

Similarly, 26 viral meningitis/encephalitis (VM) samples were compared to the control. Similarly, we identified 22 VM-vs-CTRL DEGs (Supplementary Table 3) and found 40 enriched KEGG pathways by the GSEA method. The top 10 pathways are shown in Fig. 4B, including immune rejection (hsa05330: Allograft rejection, hsa05332: Graft-versus-host disease and hsa04612: Antigen processing and presentation), and viral infections (hsa05168:Herpes simplex virus 1 infection and hsa05169: Epstein-Barr virus infection).

Finally, we tried to obtain several host genes that can distinguish BM, VM, and CTRL samples. The R package DaMiRseq was used to rank and select the most robust genes for the model (See methods), and 53 genes were obtained. The genes can separate the samples very well (Fig. 4C). And based on the 53 genes, we built a logistic regression 3-class model which showed high classification performance and achieved AUC values of 0.972, 0.967 and 0.994 for BM, VM and CTRL, respectively (Fig. 4D). The genes associated with the scores of BM and VM in the classification model can be found in Supplementary Fig. 2 and some genes are knowingly associated with infections, such as ASRGL1, NR2F6, and OLFML3 for bacterial infection (Fig. 4E) and STIP1, PGAM5, and AKAP8 for viral infections (Fig. 4F).

### Using host gene expression response to detect bacterial contaminations

**Fig. 5:**
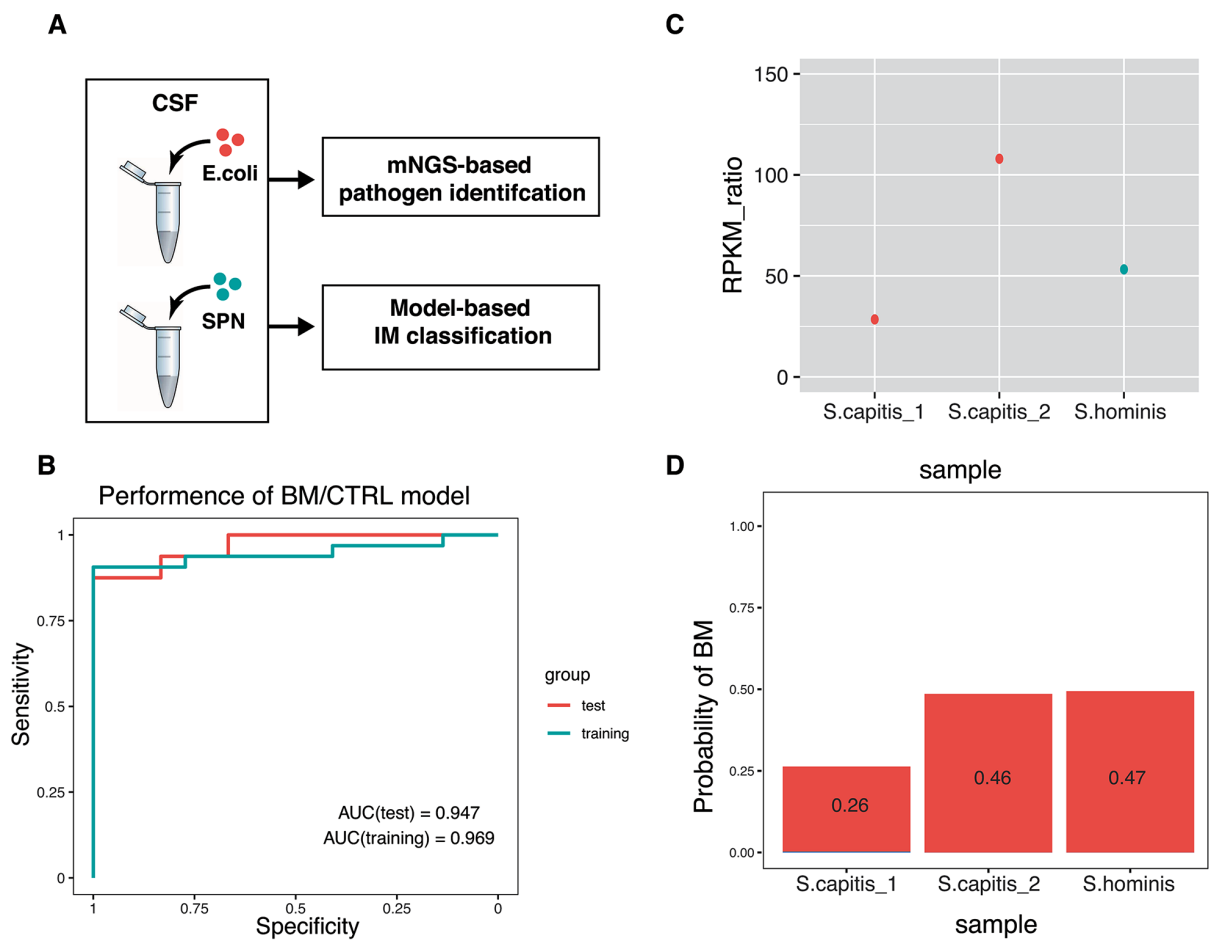
IM classification model can identify false-positive mNGS results caused by contamination. (**A**) The diagram of the host-pathogen combined method for contamination identification. (**B**) The performances of the BM/CTRL classification model. (**C**) The results of mNGS for 3 CSF samples with suspected contamination. (**D**) The results of the BM classification model for the above 3 CSF samples

Bacterial contaminations are widespread for CSF samples, leading to false-positive diagnoses and costly, possibly unnecessary treatments [36]. One strategy to identify potential contaminations is to examine host gene expression in a CSF sample because they are unlikely affected by contamination. To this end, we developed a BM/CTRL classification model based on host gene profiling (Fig. 5A).

We randomly divided 82 samples (including BM and CTRL subjects) into training (*n* = 54) and test cohorts (*n* = 28). The top five differentially expressed genes between BM and CTRL samples were selected via the DaMiRseq package (See methods) and were used to develop a logistic regression model (LRM). The model performed well in both training (AUC = 0.947, sensitivity = 90.6%, and specificity = 86.4%) and test cohorts (AUC of 0.969, sensitivity = 93.8%, and specificity = 83.3%) (Fig. 5B), providing a tool to rule out contaminations.

Candidate pathogens were identified in 3 CSF samples by mNGS reads (Fig. 5C), which may be subject to contaminations. By applying the model to these 3 CSF samples, we found that all the samples are infection-free (Fig. 5D). These results are in line with the observation that these samples are near-normal in biochemical indicators and clinical manifestations.

### Developing a model to identify BM patients with poor prognosis

**Fig. 6:**
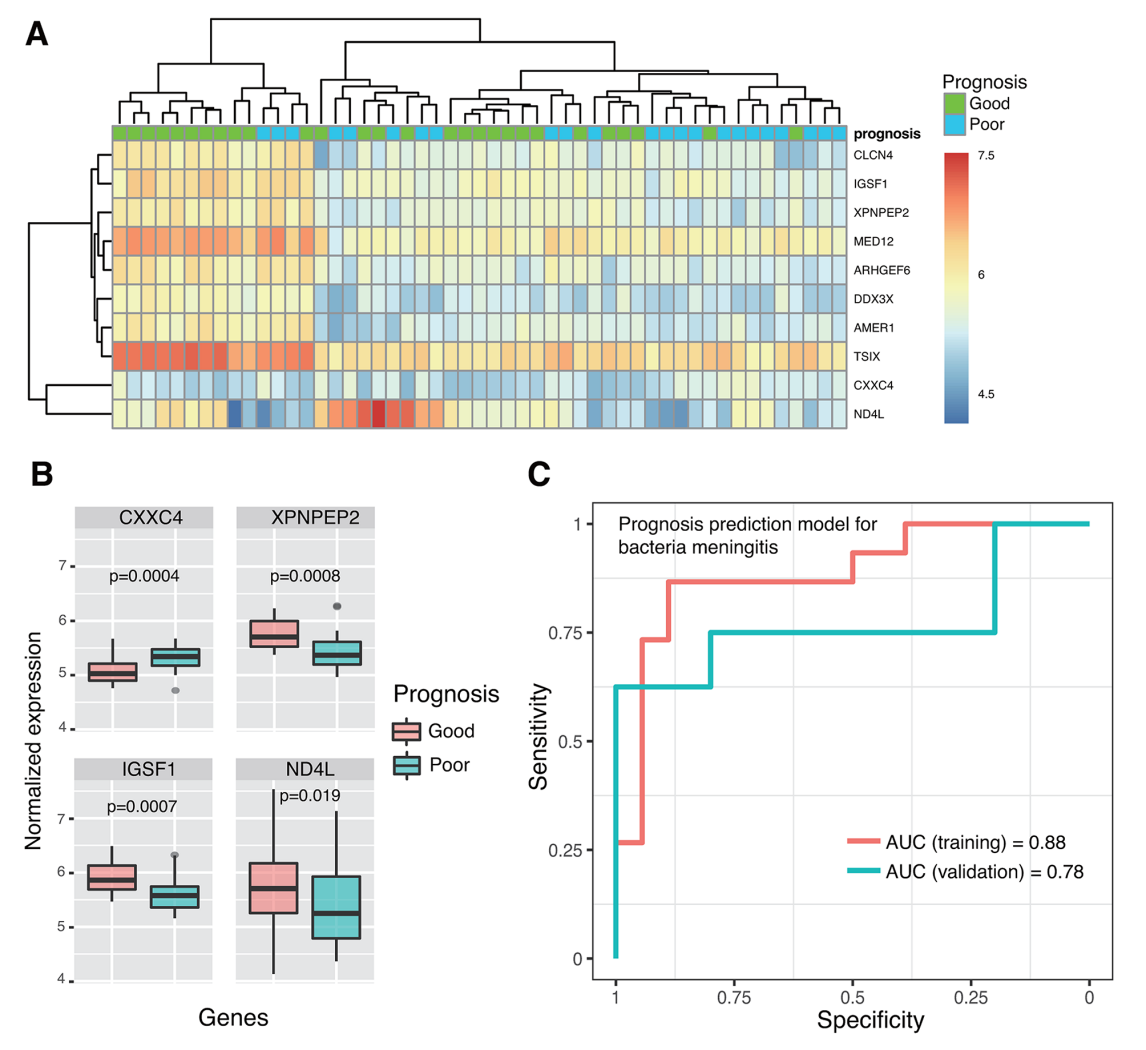
The logistics regression model for predicting BM prognosis. (**A**) The expressions of poor prognosis-related genes. (**B**) The performance of the BM prognostic risk prediction model in training and validation cohorts. (**C**) The expressions of CXXC4, XPNPEP2, IGSF1, and ND4L genes in poor and good prognosis samples

According to the outcomes when discharged, more than half of BM patients (54.9%, 28/51) had poor prognosis. Poor prognosis is associated with complications of bacterial meningitis (including subdural effusion, ependymitis, hydrocephalus, encephalomalacia, and brain abscess), withdrawal of treatment, or death [37]. To predict prognosis, we developed a model based on ten differentially expressed genes between good and poor prognosis groups selected via the DaMiRseq algorithm (Fig. 6A). The BM samples were randomly divided into training (*n* = 33) and test cohorts (*n* = 18). And a logistic regression was trained from it. Finally, four genes, including CXXC4, XPNPEP2, IGSF1 and ND4L, were used in the model (Fig. 6B). As seen in Fig. 6C, the model performs well in both training ((AUC = 0.88, sensitivity = 86.7% and specificity = 88.9%) and test cohorts (AUC = 0.78, sensitivity = 75% and specificity = 80%).

## Discussion

In this study, we reported a multifaceted mNGS-based approach for IM diagnosis, treatment, and prognosis. The approach examines two central components of CSF samples: the pathogen and host response. By combinative analyses of the pathogen and host data, our approach provides an advantage over traditional one in (1) detecting DNA and RNA pathogens simultaneously (2), identifying antibiotic resistant genes (3), pinpointing sample contamination, and (4) predicting prognosis. For example, our approach identified pathogens missed by standard clinical diagnostics, such as those in samples 48, 94, and 100.

Additionally, our approach also identified antibiotic resistant genes (ARGs), offering the potential to enhance antibiotic usage stewardship. For some commonly used antibiotics, the detected ARGs were highly consistent with resistance to antibiotics. For example, we detected the ARG blaCTX-M in two E. coli samples (6 and 133) and the samples seemed sensitive to the drug cephalosporin (Fig. 3D). Similarly, we detected ARGs ermB (ermC) and tetO (tetM) in 2 GBS samples (16 and 125) which are predicted to be resistant to Tetracycline and Macrolide (Fig. 3F). However, the identified ARGs in some cases did not match well with antibiotic testing results, which could be due to inaccurate annotations of ARGs and/or other unknown mechanisms, such as other resistance mutations; this topic needs further research [38, 39].

The host gene expression analysis also provides us another angle to diagnose IM. In particular, we identified differentially expressed genes in BM and VM samples, and these genes are associated with different functional categories. For instance, BM genes are particularly associated with oxidative stress, in line with previous reports that oxidative stress played an important role in the pathophysiology of pneumococcal meningitis [40]. Some overexpressed genes in BM samples are associated with infections, such as ASRGL1, NR2F6, and OLFML3 for bacterial infection (Fig. 4D). OLFML3 may be associated with immune responses against bacterial clearance [41]. OLFM4, a closely related member of OLFML3, could regulate proinflammatory responses to kill bacteria such as Staphylococcus aureus [42]. NR2F6 encoded a nuclear orphan receptor, which is involved in antigen-specific CD8 + memory formation after bacterial infection [43].

In contrast, VM genes are mainly enriched in immune rejection (hsa05330: Allograft rejection, hsa05332: Graft-versus-host disease and hsa04612: Antigen processing and presentation), and viral infections (hsa05168:Herpes simplex virus 1 infection and hsa05169: Epstein-Barr virus infection). Specifically, STIP1, PGAM5, and AKAP8 were over-expressed in VM samples and associated with viral infections. STIP1 could help to facilitate substrate transfer between the Hsp70 and Hsp90 molecular chaperones, which function as broad host factors for viral protein folding [44]. PGAM5 is an important regulator in antiviral responses by regulations of IFNβ production via TBK1/IRF3 signaling pathway [45]. AKAP8 was among the top pro-viral factors for SARS-CoV-2 infections [46]. These BM/VM-specific genes may be used to distinguish BM, VM, and CTRL samples, assess the likelihood of infectious meningitis, and guide empiric antimicrobials at admission.

Given the capability to examine host gene expression, our approach provides a solution to detect contamination, because in contaminated samples host responses are not expected. Our model based on BM-differentially expressed genes can detect contamination without clinical reference data and improves the accuracy of the traditional methods based on biochemistry.

By monitoring the host gene expression, we can also predict prognosis better. The existing prognostic models are mainly based on traditional clinical and laboratory indicators. For example, CSF sugar < 1 mmol/L and CSF protein > 2 g/L were reported to be independent risk factors for the poor prognosis of neonatal bacterial meningitis [47]. Five laboratory and clinical indicators (CSF culture positivity, CSF white blood cell count, hemoglobin, Glasgow Coma Scale, and pulse rate) were strongly associated with poor outcomes and used in prognosis prediction of adult bacterial meningitis with a sensitivity of 71.7% and a specificity of 63.1% [48]. We have constructed a BM prognostic prediction model using host gene response. Several genes, including CXXC4, XPNPEP2, IGSF1, and ND4L, are selected for the model via Lasso regression. CXXC4 can recruit TET2 to methylate CpG sites at promoters and CGIs in genomic DNA [10]. Pathogens can alter DNA methylation and regulate the expression and function of DNA methylation modifiers such as TETs and DNMTs, resulting in altered expression of important host genes involved in immune responses [49]. IGSF1 encodes an Ig superfamily glycoprotein on plasma membrane and can perform important functions on various immune cells [50]. XPNPEP2 may be involved in vasodilation and innate antiviral responses [51]. *ND4L* encoded a structural subunit of the mitochondrial respiratory chains. Recent findings emphasize the emerging role of the mitochondrion as a critical intracellular signaling platform regulating innate immune and inflammatory responses to pathogens [52].

## Conclusion

This study developed a comprehensive mNGS-based pipeline by simultaneously detecting two core elements of IM infections: DNA/RNA pathogen and host response. Our pipeline achieved not only accurate detection of DNA/RNA microbes but also broadened various clinical applications, including antibiotic resistance prediction, BM/VM classification, contamination detection, and prognosis prediction, with comparable cost to traditional mNGS, which may be emerging as a routine protocol for infectious meningitis. As our study is from one hospital only, it is essential to test our approach on the data from other hospitals where the spectrum of infecting organisms as well as patient demographics and health care settings may vary [53].

### Electronic supplementary material

Below is the link to the electronic supplementary material.

Supplementary Material 1. Detections of ARGs in 24 mNGS-positive CSF samples with at least 1000 reads for pathogens

Supplementary Material 2. Genes associated with the scores of BM and VM. The genes contributing to the scores of BM (A) and VM (B) in the 3-class classification model

Supplementary Material 3. The summary of sequencing reads of 142 samples

Supplementary Material 4. Identifications of microbes in the SIM cohort

Supplementary Material 5. Differentially expressed genes between IM and CTRLs

## Data Availability

All mNGS data generated in this study have been uploaded to the NCBI Sequence Read Archive (SRA) database with accession number PRJNA842783. The homemade scripts used in microbiome identification, classifier detection, and prognosis prediction are available at GitHub (https://github.com/ScenXing/IM_research).

## Abbreviations

CIM: Confirmed infectious meningitis/encephalitis
SIM: Suspected infectious meningitis/encephalitis
CTRL: Noninfectious controls
CSF: Cerebrospinal fluid
CNS: Central nervous system
NCSF: Negative cerebrospinal fluid
KEGG: Kyoto Encyclopedia of Genes and Genomes
mNGS: Metagenomic next-generation sequencing
c-mNGS: Comprehensive mNGS
SP: Streptococcus pneumoniae
E.coli: Escherichia coli
AB: Acinetobacter baumannii
GBS: Group B Streptococcus
ESBL: Extended-Spectrum β-lactamase

## References

[1] Shen H (2019). The etiology of acute meningitis and encephalitis syndromes in a sentinel pediatric hospital, Shenzhen, China. BMC Infect Dis.

[2] Kim KS (2015). Neonatal bacterial meningitis. Neoreviews.

[3] Galiza EP, Heath PT. Improving the outcome of neonatal meningitis. *Current Opinion in Infectious Diseases* Preprint at 10.1097/QCO.0b013e32832ad49e (2009). 10.1097/QCO.0b013e32832ad49e 19333122

[4] Han D (2019). mNGS in clinical microbiology laboratories: on the road to maturity. Crit Rev Microbiol.

[5] Wilson MR (2014). Actionable diagnosis of Neuroleptospirosis by Next-Generation sequencing. N Engl J Med.

[6] Xing XW et al. Metagenomic next-generation sequencing for diagnosis of infectious encephalitis and meningitis: a large, prospective Case Series of 213 patients. Front Cell Infect Microbiol 10, (2020). 10.3389/fcimb.2020.00088 PMC7066979 32211343

[7] Hasan MR (2020). A metagenomics-based diagnostic approach for central nervous system infections in hospital acute care setting. Sci Rep.

[8] Parker J, Chen J (2017). Application of next generation sequencing for the detection of human viral pathogens in clinical specimens. J Clin Virol.

[9] van Boheemen S (2020). Retrospective validation of a metagenomic sequencing protocol for combined detection of RNA and DNA viruses using respiratory samples from Pediatric patients. J Mol Diagn.

[10] Bal A (2018). Quality control implementation for universal characterization of DNA and RNA viruses in clinical respiratory samples using single metagenomic next-generation sequencing workflow. BMC Infect Dis.

[11] Jiang H, et al. Comparison and development of a metagenomic next generation sequencing protocol for combined detection of DNA and RNA pathogens in cerebrospinal fluid. BMC Infect Dis. 2022;1–10. 10.1186/s12879-022-07272-y. 10.1186/s12879-022-07272-y PMC8976360 35365081

[12] Langelier C et al. Metagenomic sequencing detects respiratory pathogens in hematopoietic cellular transplant patients. *American Journal of Respiratory and Critical Care Medicine* Preprint at 10.1164/rccm.201706-1097LE (2018). 10.1164/rccm.201706-1097LE PMC5821905 28686513

[13] Langelier C et al. Metagenomic Next-Generation Sequencing Detects Pulmonary Pathogens in Hematopoietic Cellular Transplant Patients with Acute Respiratory Illnesses. *bioRxiv* (2017).

[14] Standage SW, Wong HR. Biomarkers for pediatric sepsis and septic shock. *Expert Review of Anti-Infective Therapy* Preprint at 10.1586/eri.10.154 (2011). 10.1586/eri.10.154 PMC3033193 21171879

[15] Briassoulis G, Galani A. Prognostic markers of pediatric meningococcal sepsis. *Expert Review of Anti-Infective Therapy* Preprint at 10.1586/14787210.2014.945431 (2014). 10.1586/14787210.2014.945431 25088380

[16] Jong VL (2016). Transcriptome assists prognosis of disease severity in respiratory syncytial virus infected infants. Sci Rep.

[17] Langelier C (2018). Integrating host response and unbiased microbe detection for lower respiratory tract infection diagnosis in critically ill adults. Proc Natl Acad Sci U S A.

[18] Ramachandran PS (2022). Integrating central nervous system metagenomics and host response for diagnosis of tuberculosis meningitis and its mimics. Nat Commun.

[19] Wood DE, Lu J, Langmead B (2019). Improved metagenomic analysis with Kraken 2. Genome Biol.

[20] Bolger AM, Lohse M, Usadel B, Trimmomatic (2014). A flexible trimmer for Illumina sequence data. Bioinformatics.

[21] Breitwieser FP, Salzberg SL, Pavian (2020). Interactive analysis of metagenomics data for microbiome studies and pathogen identification. Bioinformatics.

[22] Yin X et al. ARGs-OAP v2.0 with an expanded SARG database and Hidden Markov Models for enhancement characterization and quantification of antibiotic resistance genes in environmental metagenomes. in *Bioinformatics* (2018). 10.1093/bioinformatics/bty053. 10.1093/bioinformatics/bty053 29408954

[23] Yang Y, Li B, Ju F, Zhang T (2013). Exploring variation of antibiotic resistance genes in activated sludge over a four-year period through a metagenomic approach. Environ Sci Technol.

[24] Dobin A, et al. Ultrafast universal RNA-seq aligner. Bioinformatics. 2013;STAR. 10.1093/bioinformatics/bts635. 10.1093/bioinformatics/bts635 PMC3530905 23104886

[25] Liao Y, Smyth GK, Shi W, FeatureCounts (2014). An efficient general purpose program for assigning sequence reads to genomic features. Bioinformatics.

[26] Love MI, Huber W, Anders S (2014). Moderated estimation of Fold change and dispersion for RNA-seq data with DESeq2. Genome Biol.

[27] Yu G, Wang LGG, Han Y, He QYY, ClusterProfiler (2012). An R package for comparing biological themes among gene clusters. OMICS.

[28] Chiesa M, Colombo GI, Piacentini L (2018). DaMiRseq -An R/Bioconductor package for data mining of RNA-Seq data: normalization, feature selection and classification. Bioinformatics.

[29] Friedman J, Hastie T, Tibshirani R (2010). Regularization paths for generalized linear models via coordinate descent. J Stat Softw.

[30] Robin X (2011). pROC: an open-source package for R and S + to analyze and compare ROC curves. BMC Bioinformatics.

[31] Sing T, Sander O, Beerenwinkel N, Lengauer T (2005). ROCR: visualizing classifier performance in R. Bioinformatics.

[32] Wilson MR (2019). Clinical metagenomic sequencing for diagnosis of Meningitis and Encephalitis. N Engl J Med.

[33] Su M, Satola SW, Read TD (2019). Genome-based prediction of bacterial antibiotic resistance. J Clin Microbiol Preprint at.

[34] Langelier C (2019). Microbiome and antimicrobial resistance gene dynamics in international travelers. Emerg Infect Dis.

[35] Ellington MJ et al. The role of whole genome sequencing in antimicrobial susceptibility testing of bacteria: report from the EUCAST Subcommittee. *Clinical Microbiology and Infection* Preprint at 10.1016/j.cmi.2016.11.012 (2017). 10.1016/j.cmi.2016.11.012 27890457

[36] Boysen MM, Henderson JL, Rudkin SE, Burns MJ, Langdorf MI (2009). Positive cerebrospinal fluid cultures after normal cell counts are contaminants. J Emerg Med.

[37] Liu M, Di (2019). Risk factors for poor prognosis of neonatal bacterial meningitis. Chin J Contemp Pediatr.

[38] Blair JMA, Webber MA, Baylay AJ, Ogbolu DO, Piddock L (2015). J. V. Molecular mechanisms of antibiotic resistance. Nat Reviews Microbiol Preprint at.

[39] Peterson E, Kaur P (2018). Antibiotic resistance mechanisms in bacteria: relationships between resistance determinants of antibiotic producers, environmental bacteria, and clinical pathogens. Front Microbiol.

[40] Barichello T, Generoso JS, Simões LR, Elias SG, Quevedo J. Role of oxidative stress in the pathophysiology of pneumococcal meningitis. *Oxidative Medicine and Cellular Longevity* Preprint at 10.1155/2013/371465 (2013). 10.1155/2013/371465 PMC3665263 23766853

[41] Toedebusch RG (2021). Microglia-derived olfactomedin-like 3 promotes pro-tumorigenic microglial function and malignant features of glioma cells. Int J Mol Sci.

[42] Liu W (2013). Olfm4 deletion enhances defense against Staphylococcus aureus in chronic granulomatous disease. J Clin Invest.

[43] Jakic B (2021). Loss of the orphan nuclear receptor NR2F6 enhances CD8 + T-cell memory via IFN-γ. Cell Death Dis.

[44] Bhattacharya K (2020). The Hsp70-Hsp90 co-chaperone Hop/Stip1 shifts the proteostatic balance from folding towards degradation. Nat Commun.

[45] Yu Y, qiang (2020). PGAM5-MAVS interaction regulates TBK1/ IRF3 dependent antiviral responses. Sci Rep.

[46] Flynn RA (2021). Discovery and functional interrogation of SARS-CoV-2 RNA-host protein interactions. Cell.

[47] Liu M, Di (2019). Risk factors for poor prognosis of neonatal bacterial meningitis. Chin J Contemp Pediatr.

[48] Wall EC (2017). Prediction of outcome from adult bacterial meningitis in Ta high-HIV-seroprevalence, resource-poor setting using the Malawi adult meningitis score (MAMS). Clin Infect Dis.

[49] Qin W, Scicluna BP, van der Poll T. The Role of Host Cell DNA Methylation in the Immune Response to Bacterial Infection. *Frontiers in Immunology* Preprint at 10.3389/fimmu.2021.696280 (2021). 10.3389/fimmu.2021.696280 PMC8358789 34394088

[50] Joustra SD (2013). IGSF1 deficiency syndrome. Rare Dis.

[51] Menicucci AR, Jankeel A, Feldmann H, Marzi A, Messaoudi I (2019). Antiviral innate responses induced by VSV-EBOV vaccination contribute to rapid protection. mBio.

[52] Jin HS, Suh HW, Kim SJ, Jo EK. Mitochondrial control of innate immunity and inflammation. *Immune Network* Preprint at 10.4110/in.2017.17.2.77 (2017). 10.4110/in.2017.17.2.77 PMC5407986 28458619

[53] Taylor JMG, Ankerst DP, Andridge RR (2008). Validation of biomarker-based risk prediction models. Clin Cancer Res Preprint at.

